# Thermo- and electro-optical properties of photonic liquid crystal fibers doped with gold nanoparticles

**DOI:** 10.3762/bjnano.8.278

**Published:** 2017-12-27

**Authors:** Agata Siarkowska, Miłosz Chychłowski, Daniel Budaszewski, Bartłomiej Jankiewicz, Bartosz Bartosewicz, Tomasz R Woliński

**Affiliations:** 1 Faculty of Physics, Warsaw University of Technology, Koszykowa 75, 00-662, Warsaw, Poland; 2 Institute of Optoelectronics, Military University of Technology, Kaliskiego 2, 00-908, Warsaw, Poland

**Keywords:** fiber optics, gold nanoparticle, liquid crystal, phase transition temperature, photonic crystal fiber

## Abstract

Thermo- and electro-optical properties of a photonic liquid crystal fiber (PLCF) enhanced by the use of dopants have been investigated. A 6CHBT nematic liquid crystal was doped with four different concentrations of gold nanoparticles (NPs), 0.1, 0.3, 0.5 and 1.0 wt %, for direct comparison of the influence of the dopant on the properties of the PLCF. The thermo-optical effects of the liquid crystal doped with gold NPs were compared in three setups, an LC cell, a microcapillary and within the PLCF, to determine if the observed responses to external factors are caused by the properties of the infiltration material or due to the setup configuration. The results obtained indicated that with increasing NP doping a significant reduction of the rise time under an external electric field occurs with a simultaneous decrease in the nematic–isotropic phase transition temperature, thus improving the thermo- and electro-optical properties of the PLCF.

## Introduction

Since their discovery in 1888, liquid crystals (LCs) have attracted nonstop research interest for their unique electro-optical properties that are essential in various optical and photonic applications such as LC displays, attenuators, tunable polarizers, spatial light modulators, photonic sensors, etc. [1–2]. However, current values of electro-optic response times and switch-on voltages still could be (and for some applications need to be) improved. To accomplish this, LCs are being either stabilized by polymers [3] or doped with special materials such as nanotubes [4] and quantum dots [5]. Over the last few years nanoparticles (NPs) have been used to improve the electro-optics properties of LCs as they can influence, for example, the LC response time and decrease the Freedericksz threshold voltage [6–8]. Promising results were reported for metallic (mostly gold and silver), ferroelectric and dielectric NPs [9–12]. It appeared that NP/LC molecule size matching can strongly influence the intrinsic properties of the NP-doped LC [13]. However, the use of such dopants also comes with new difficulties. One of the crucial issues is NP aggregation, especially in mixtures with higher concentrations of NPs. At low concentrations (below 1 wt %), diluted NP suspensions will be stable due to relatively weak interactions of dopant particles [14].

Up to now, NP-doped LCs have been reported mostly in LC cells. In this paper, we present the experimental results of electro- and thermo-optic properties of LC cells as well as silica glass microcapillaries (MCs) and photonic crystal fibers (PCFs) infiltrated with a NP-doped nematic LC. PCFs infiltrated with LCs (Larsen et al. [15]) have been known since 2005 [16] as photonic liquid crystal fibers (PLCFs). PLCFs offer a high level of tunability due to significantly improved control of their spectral, polarizing, and guiding properties. We strongly believe that the presence of metallic NPs could provide even greater improvement to the electro-optical parameters of PLCFs. First attempts to infiltrate a PCF with LCs doped with barium titanate NPs were reported in 2009 [17], providing new features such as frequency modulation response or a transmission spectrum with tunable attenuation. Another NP material used in PLCFs is titanium. It has been demonstrated that the doping concentration of Ti NPs in a nematic LC influences its electro-optic properties. The first experimental evidence of improved thermo-optic properties of a PLCF with a Ti-doped 5CB nematic LC has been recently reported in [18], where a noticeable difference in both the orientation of the molecules and the propagation spectra of the PLCF was observed. The light was guided according to a mechanism known as the photonic bandgap (PBG) effect, in which only selected wavelengths are propagated in the core region, and the refractive index of the core is lower than in the cladding region. Preliminary results indicate that the observed PBG materials can be temperature-tuned towards smaller wavelengths. However, the Ti-doped LC cells did not reveal any improvement of the response time under an external electric field.

**Figure 1:**
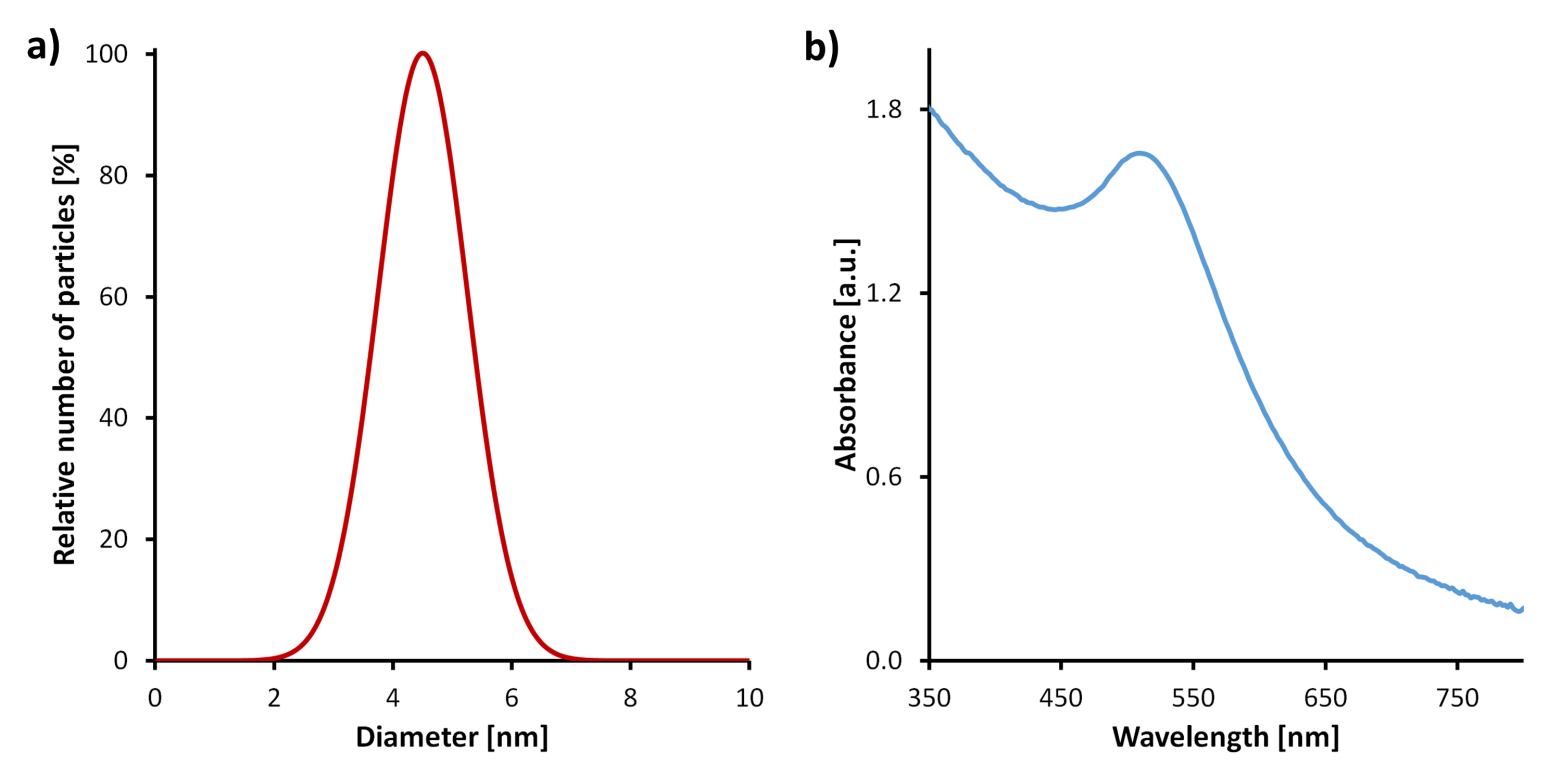
Properties of the Au nanoparticles (NPs): particle size distribution of Au NPs obtained by using a disc centrifuge sedimentation method (a) and UV–vis spectrum of Au NPs in an organic solvent (b).

In the present research we used a 6CHBT nematic LC [19] and 4 nm diameter, spherical Au NPs (Figure 1a), both synthesized in the Military University of Technology, Warsaw, Poland. Au NPs have been used for applications both in photonics and biology [20] due to their optical properties. These properties are related to the interaction of light with electrons on the NP surface. At a specific frequency of light, the oscillation of electrons on the Au NP surface causes an effect called localized surface plasmon resonance. This phenomena can result in absorption or scattering of light (Figure 1b). Depending on the size, concentration or shape of the particles, the plasmon resonance can appear at different wavelengths. Moreover, NPs can also provide different properties for the host material. For example, Au NPs have a tendency to lower the nematic–isotropic phase transition temperature [21–22], but with a specific surface coating, the effect can be reversed [23]. The presence of Au NPs in an LC has proven to influence the response time and lower the threshold voltage [24–28].

In this paper we compare four different concentrations of gold NPs (0.1, 0.3, 0.5 and 1 wt %) mixed with a 6CHBT nematic LC. These mixtures were tested in LC cells, microcapillaries and PLCFs to observe the influence of the structure geometry on the sample response to external factors such as temperature and electric field. To the best of our knowledge there are no published reports considering a PLCF with Au-doped LCs.

## Experimental

The experiments were divided into two parts: the investigation of the temperature influence on Au NP-doped LCs (with a focus on the nematic–isotropic (N–I) phase transition temperature) and the influence of an external electric field (with a focus on switching time). In the first part, different host structures were used: i) a LC cell, ii) microcapillaries, and iii) a photonic crystal fiber, in order to compare the influence of a NP-doped LC in setups with different geometries. This also allowed us to specify if the observed response to external factors is caused by the properties of the infiltrated material or due to the setup configuration. In the second part of the experiments, we focused only on PLCFs doped with gold NPs. The LC material used for infiltration of the host structure was a nematic 4-(trans-4'-*n*-hexylcyclohexyl)isothiocyanatobenzene (6CHBT) LC, which has the following parameters at room temperature: *n*_e_ = 1.67, *n*_o_ = 1.52, Δ*n* = 0.15 and Δε = 8.0. The other parameters of this material can be found in [19]. Before infiltration into the structure, the NP-doped LC was sonicated and heated above the N–I transition temperature. This technique allowed us to obtain a uniform mixture without any sedimentation, which could plug the microchannels of the PCF and hinder (or even prevent) the infiltration process.

### Synthesis of liquid crystal–Au nanoparticle composites

The Au NP-doped LC mixture was fabricated by using a modification of the method reported elsewhere [29]. Au NPs were synthesized by borohydride reduction of gold salts in water. To 10 mL of the Au NP colloidal solution 5 mL of chloroform and 20 µL of 1-dodecanethiol (DDT) were added. A hydrophobic shell on the NP surface was formed by DDT molecules, which induced a phase transfer of the Au NPs from water to chloroform. The LC–Au NP composites with various Au NP concentrations (0.1, 0.3, 0.5 and 1 wt %) were fabricated by mixing the suspension of Au NPs in chloroform with a liquid crystal material and slowly evaporating the solvent.

### Nematic–isotropic phase transition temperature: liquid crystal cells and microcapillaries

Initial experiments were done in an LC cell with a thickness of 5 µm as well as with MCs with an inner diameter of 20 µm and about 15 cm length. The Au-doped LCs were filled into the setup structure in the isotropic phase due to capillary forces and after that they were put on the computer-controlled hot stage (Linkam) and slowly heated (at rate 0.1 °C/min) above the phase transition temperature. The observed results were registered under a microscope with crossed polarizers (Nikon Eclipse).

### Nematic–isotropic phase transition temperature: the photonic crystal fiber

**Figure 2:**
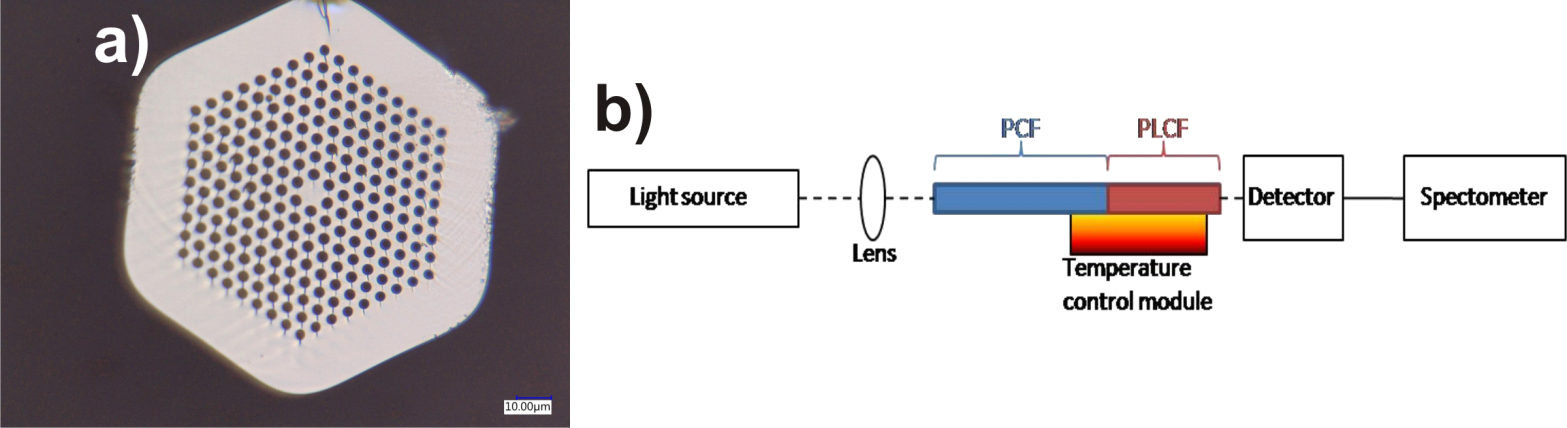
Cross-section of the PCF 061221 (a) and experimental setup for measuring the propagation spectra in PLCF (b).

Next, we introduced the NP-doped LC into a PCF (cat. no. 061221, manufactured in UMCS, Lublin, Poland) with an outer diameter of 135 µm, hole diameter of 4.1 µm and lattice constant of about 6.5 µm (Figure 2a). Similarly to as previously described, the NP-doped LC mixture was heated above the 6CHBT N–I phase transition temperature and then introduced into the PCF by capillary force. Unlike in previous experiments, where the sample was observed under a polarizing microscope, here we focused on the light exiting the fiber core. The preliminary part of the experiment was focused on the comparison of the propagation spectra of the PLCFs with different infusions at room temperature. The experimental setup is shown in Figure 2b, in which we used a halogen lamp (Mikropak HL-2000) as a light source and the output spectra were recorded by a fiber optic spectrometer (Ocean Optics, USB4000).

### Setup for experiments in an electric field

**Figure 3:**
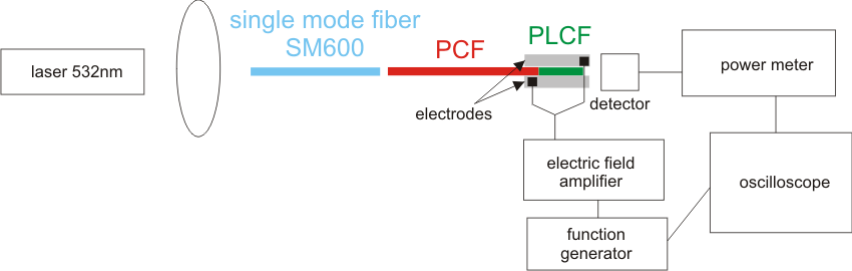
Experimental setup for measuring changes of the switching times under an external electric field.

The experimental setup is shown in Figure 3. We used a green laser operating at a wavelength of 532 nm as a light source in this experiment due to the fact that at this wavelength all the samples (observed in the first part of the experiment) had relatively good propagation properties. Light was first injected into a single-mode SM 600 fiber and coupled face-to-face with the PCF that was infiltrated partially with a 6CHBT LC forming the PLCF. The infiltrated part of the fiber was placed between two parallel, flat electrodes separated with two additional fibers on the sides with the same diameter as the measured fiber. These additional fibers were glued to one of two electrodes and they acted as spacers to maintain height control. There was a layer of silicon between the electrodes to prevent electric discharge that could destroy the sample. More details of the experimental setup are presented elsewhere [30]. The output signal was collected by a Newport power meter. The electrodes were connected to the voltage generator and were controlled in a range of 1.6–11.2 V/µm (with 0.2 V/µm step) with a rectangular signal at 1 kHz frequency and amplitude modulation of 5 Hz. The definition of switching time was 10–90% and the uncertainty of the measurements is about 2 ms.

## Results and Discussion

### Nematic–isotropic phase transition temperature

**Figure 4:**
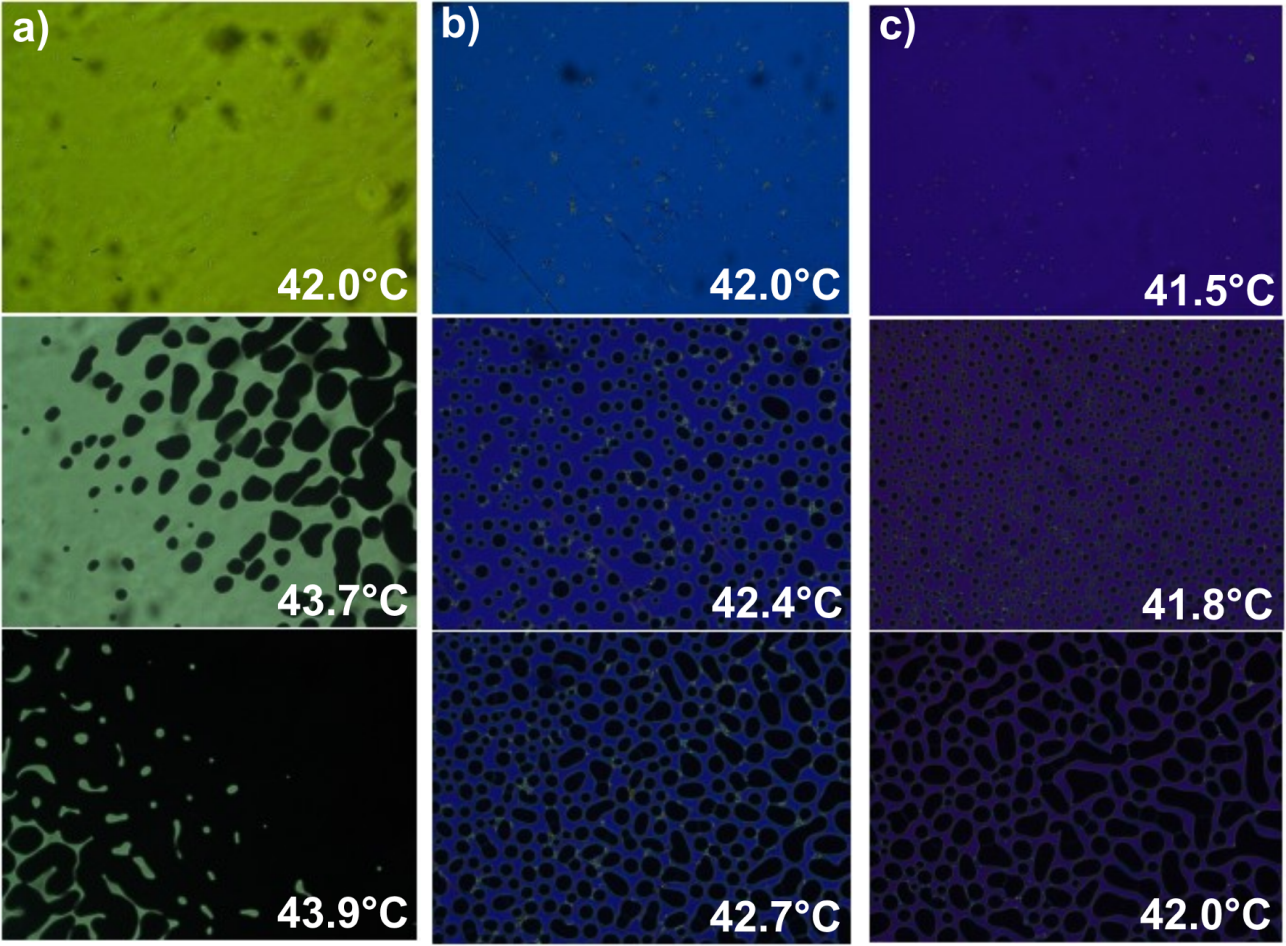
Liquid crystal (LC) cells filled with (a) undoped, (b) 0.5 wt % Au-doped and (c) 1 wt % Au-doped 6CHBT LC.

**Figure 5:**
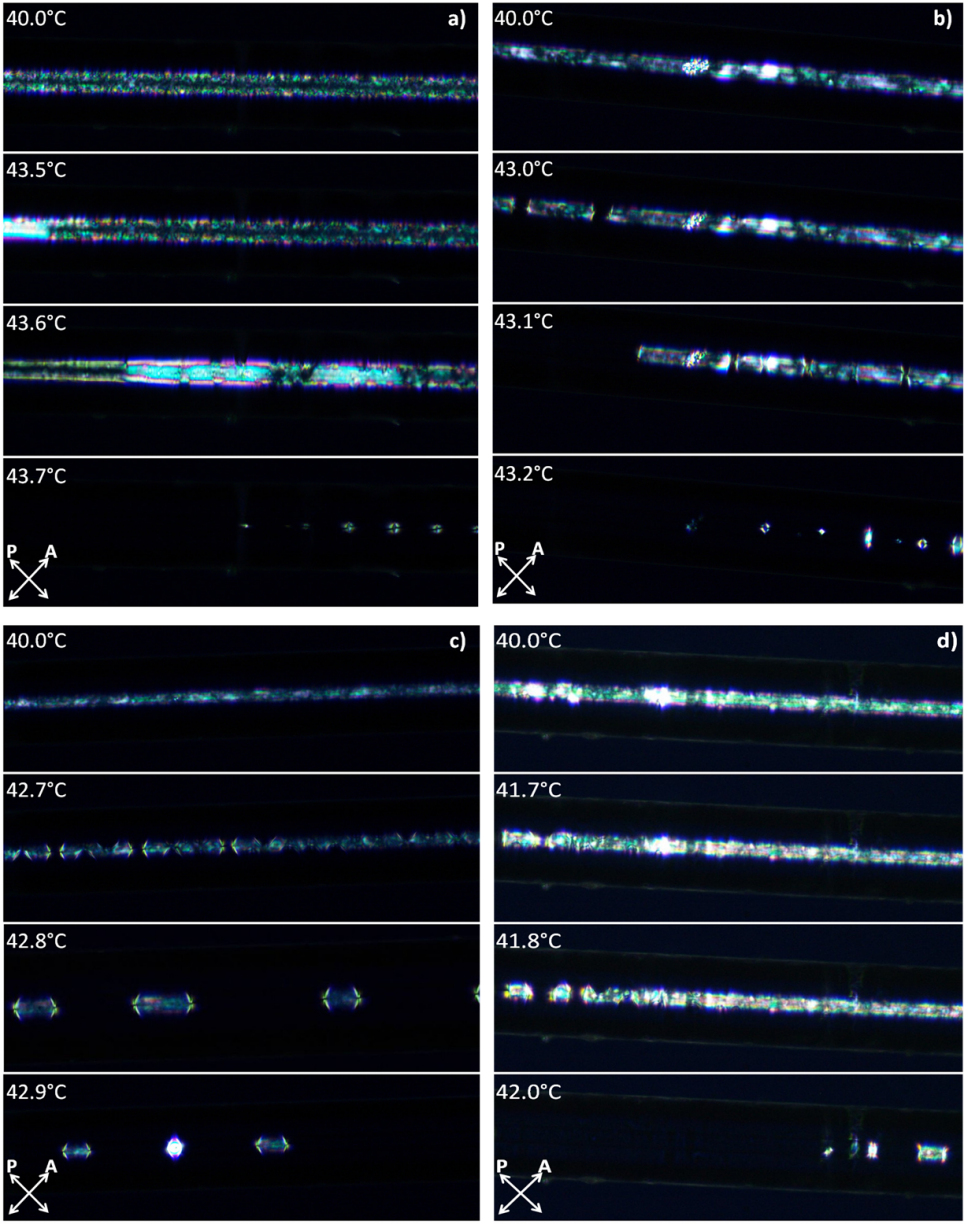
Microcapillaries infiltrated with (a) undoped, (b) 0.3 wt % Au-doped, (c) 0.5 wt % Au-doped and (d) 1 wt % Au-doped 6CHBT LC.

As reported elsewhere [22–23], Au NPs have a tendency to reduce the N–I phase transition temperature of the LC. To verify this, experiments were performed in three different setups to eliminate the possibility that any change in the N–I phase transition temperature is related to the specific setup. For LC cells, the N–I phase transition temperature was taken as the temperature where isotropic droplets appeared in the sample. Figure 4 shows the behavior of undoped and Au-doped (0.5 wt % and 1 wt %) LC cells at a temperature near the phase transition. For the MCs the N–I phase transition temperature was defined as the temperature where the nematic phase begins to become distorted and slowly transitions to the isotropic phase. In Figure 5 we show the behavior of undoped and Au-doped (0.3, 0.5 and 1 wt %) LCs inside a MC near the phase transition temperature. For all investigated samples, planar orientation was observed, with maximum light intensity for the sample aligned at 45° relative to the polarizers.

Similar effects were observed for both LC cells and MCs. We observed that an increase of the Au dopant in the LC lowered the N–I phase transition temperature. For lower NP concentrations (0.1 wt % and 0.3 wt %), the transition temperature remained close to 43 °C. However, a higher concentration noticeably lowered the phase transition temperature. For example, in the MCs for an undoped LC the N–I transition process started at 43.5 °C, and after adding NPs, the temperature decreased to 43.3 °C (0.1 wt %), 43.0 °C (0.3 wt %), 42.7 °C (0.5 wt %), and 41.7 °C (1 wt %), respectively.

**Figure 6:**
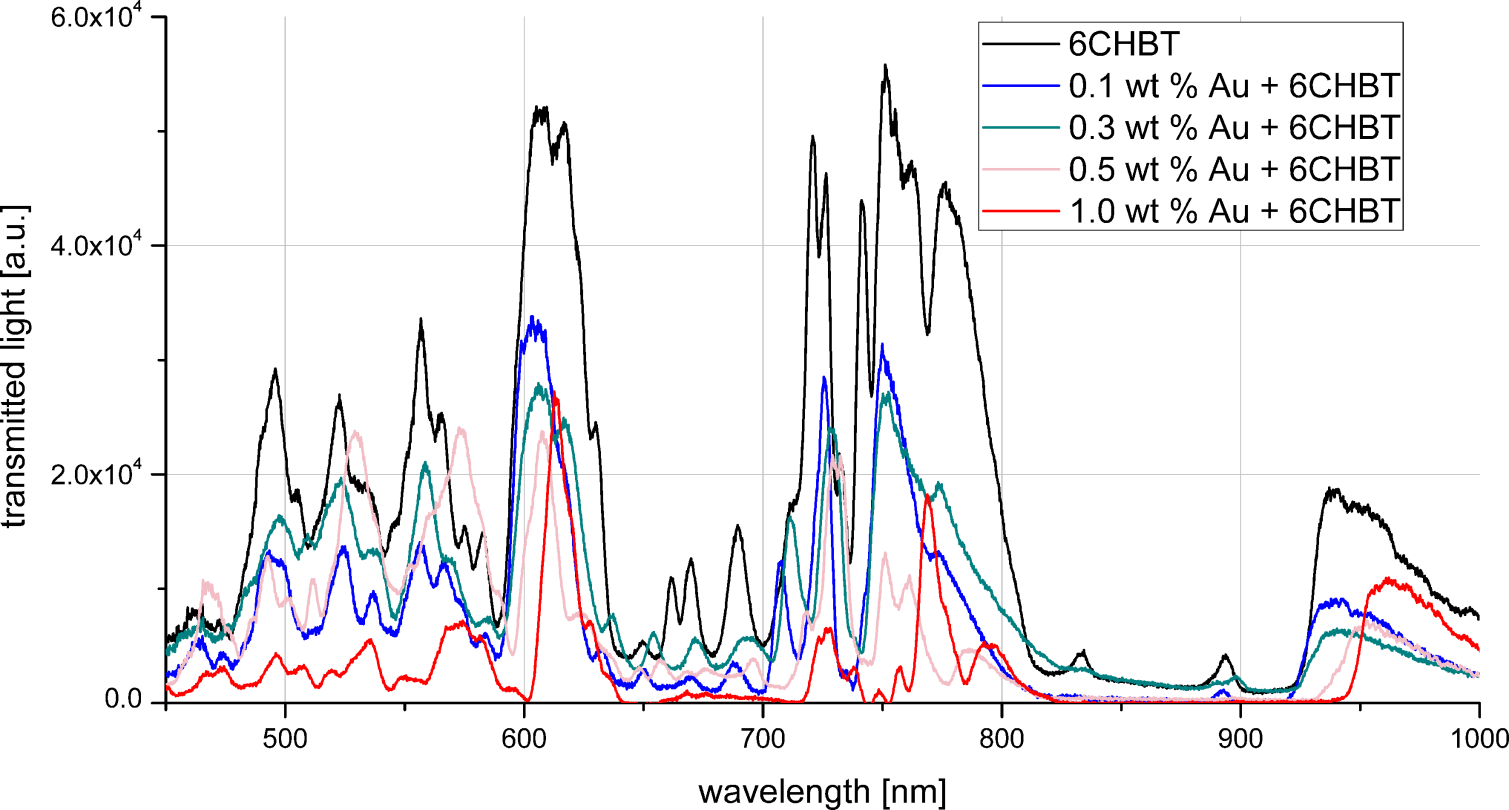
Propagation spectra for a photonic liquid crystal fiber (PLCF) infiltrated with 6CHBT LC (black) at 0.1 wt % (blue), 0.3 wt % (green), 0.5 wt % (pink) and 1 wt % Au doping 6CHBT (red) at room temperature.

After that, we introduced the NP-doped LC into a PCF, creating the so-called NP-PLCF. As shown in Figure 6, three strong and several weaker maxima were observed. The first and strongest peak was registered in the wavelength range of 600–650 nm and two other peaks were registered for 700–730 nm and 750–780 nm. The weak peaks are on the edges of the observed spectrum: 550–600 nm and 900–950 nm. We have observed that the spectra of the PLCFs with NP-doped 6CHBT nematic LCs are generally shifted towards longer wavelengths and the shift is more visible for a Au NP concentration higher than 0.3 wt % (Figure 6). The shift for the 1 wt % concentration is about 10–15 nm, whereas for the lowest concentration it is below 3 nm. The spectrum shift characterizing the NP-doped PLCF might be attributed to the presence of metallic NPs that introduce orientation distortions around LC molecules. Some authors [31–32] explain such a behavior in terms of plasmon resonance tuning, i.e., NPs absorbing at a selective wavelength can heat LC molecules, thus shifting the propagation spectrum. A similar effect can be observed in PLCFs, but the mechanism of PBG tuning was thermally induced [33]. However, we believe that the observed spectra shifts are not directly related to plasmon resonance tuning. The excitation source was a halogen lamp, which produces light of constant amplitude, but the intensity of the light coupled into the PLCF is low. The majority of the intensity of the propagating modes remains in the core region of the PLCF and the remaining part is coupled to the cladding. For this reason, the interaction of the propagating light in the fiber having a photonic structure with the NP-doped LC is too small for plasmon resonance to induce thermal tuning. Apart from the spectra shift, we have observed a reduction of the transmitted light intensity, in particular at shorter wavelengths.

**Figure 7:**
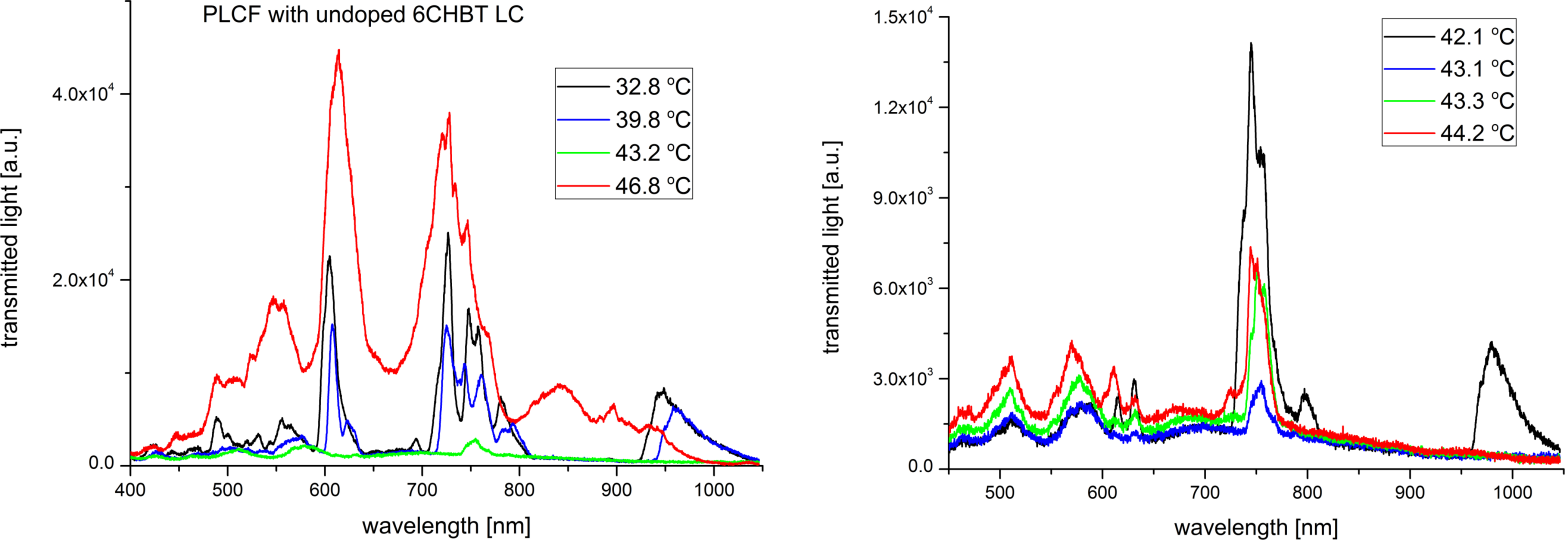
Thermal spectra of propagation in a photonic liquid crystal fiber filled with undoped liquid crystal. Spectra up to the phase transition temperature (left) and magnification of the nematic–isotropic phase transition point (right).

**Figure 8:**
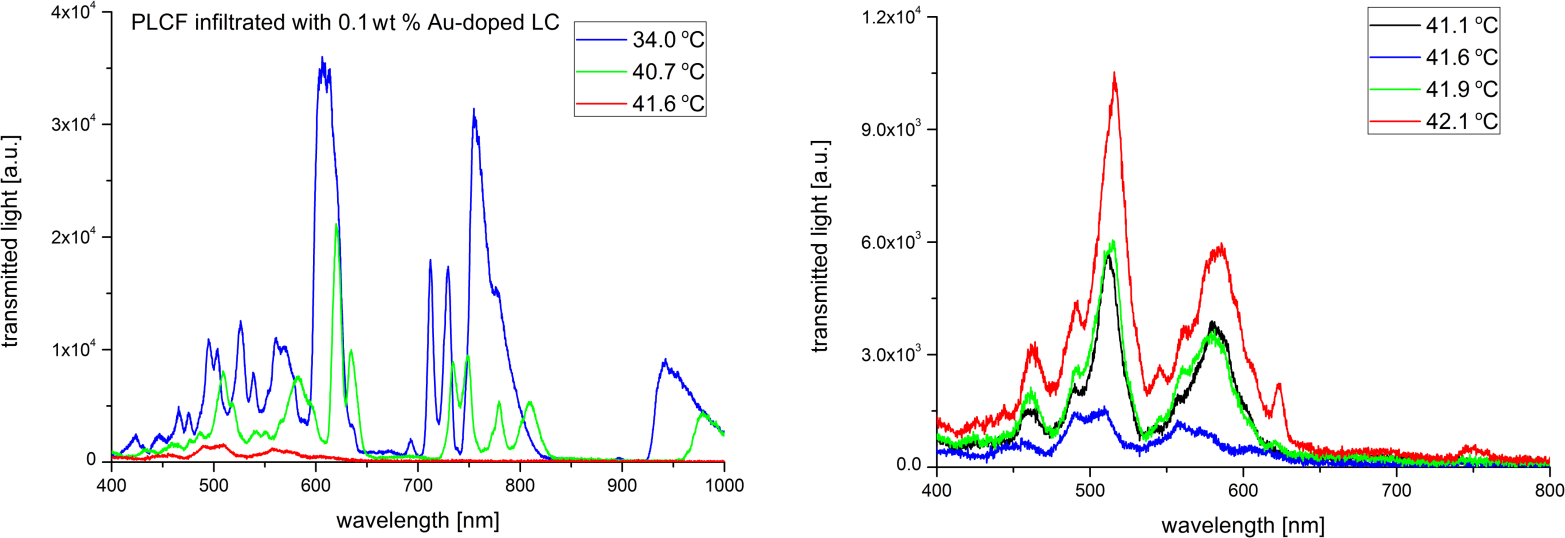
Thermal spectra of propagation in a photonic liquid crystal fiber filled with 0.1 wt % Au-doped liquid crystal. Spectra up to the phase transition temperature (left) and magnification of the nematic–isotropic phase transition point (right).

**Figure 9:**
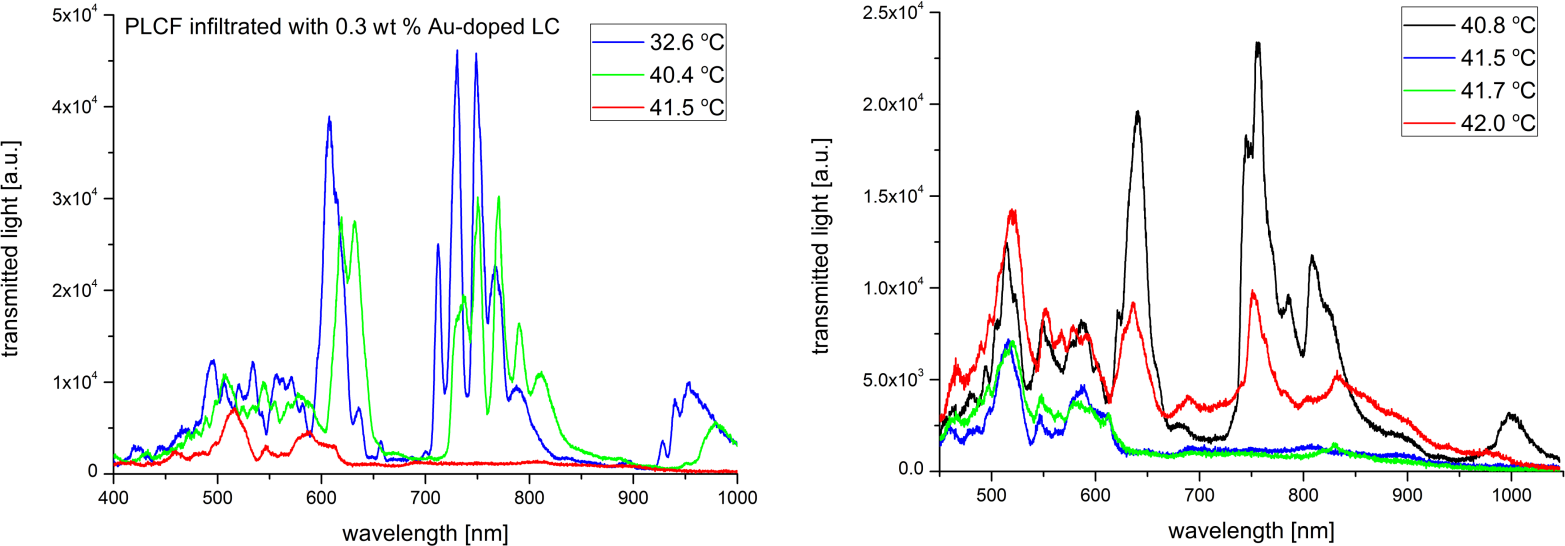
Thermal spectra of propagation in a photonic liquid crystal fiber filled with 0.3 wt % Au-doped liquid crystal. Spectra up to the phase transition temperature (left) and magnification of the nematic–isotropic phase transition point (right).

**Figure 10:**
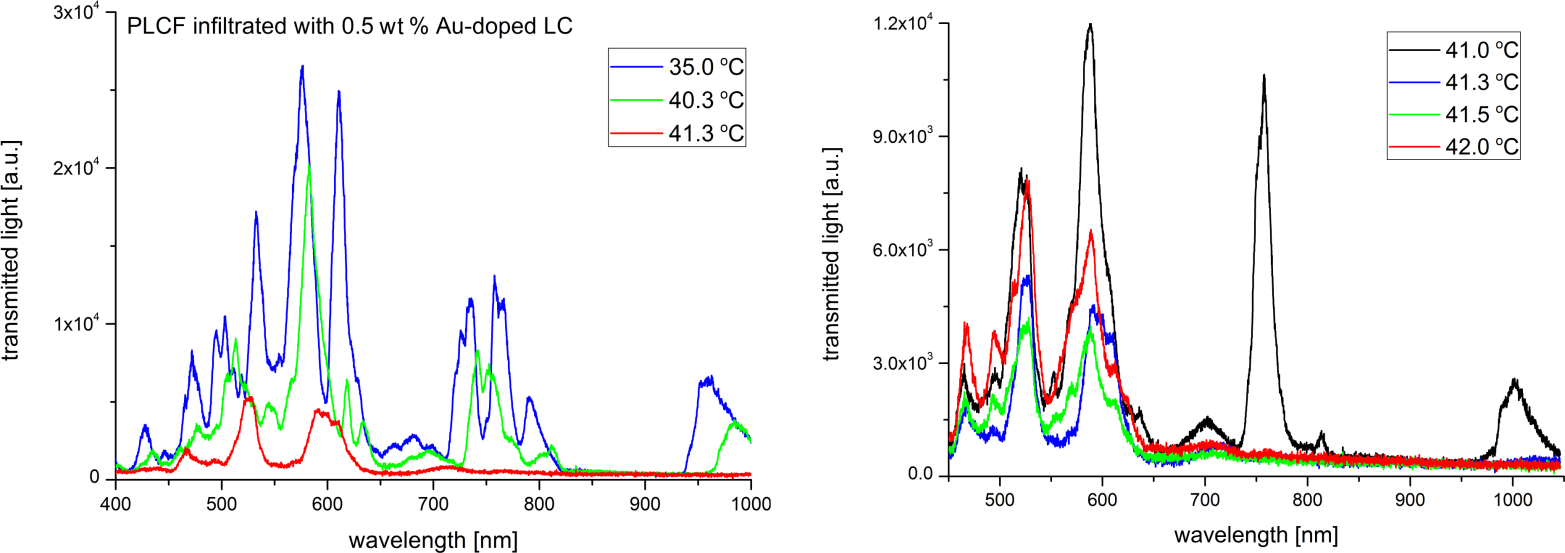
Thermal spectra of propagation in a photonic liquid crystal fiber filled with 0.5 wt % Au-doped liquid crystal. Spectra up to the phase transition temperature (left) and magnification of the nematic–isotropic phase transition point (right).

**Figure 11:**
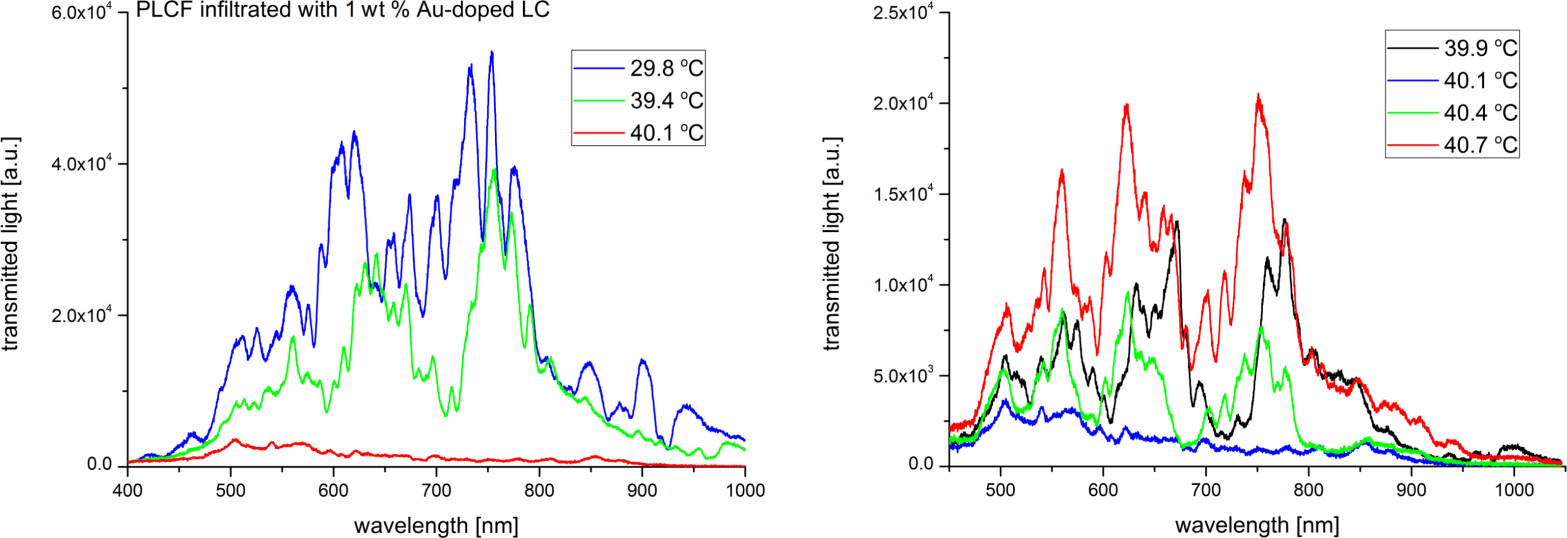
Thermal spectra of propagation in a photonic liquid crystal fiber filled with 1 wt % Au-doped liquid crystal. Spectra up to the phase transition temperature (left) and magnification of the nematic–isotropic phase transition point (right).

In order to investigate the thermal effects we have implemented the Peltier module right under the infiltrated part of the PLCF (see Figure 2) and light propagation spectra up to the N–I phase transition temperature were recorded. Figures 7–11 show the spectra of the PLCFs with both undoped and doped LCs with four different NP concentrations. In all these cases, an increase of temperature up to about 40 °C resulted in a "red shift" related to decreased light transmission.

It has been observed that the flattening of the recorded spectra (which is an effect of the N–I phase transition) took place at different temperatures. The obtained N–I phase transition temperatures are shown in Table 1 and compared with the temperatures for MCs and LC cells. It can be noticed that the results for PLCFs have lower temperatures than for the other two setups. This can be attributed to a different observation method used for PLCFs. However, for all three setup materials, there is a similar tendency that the NP-doped LC reduces the N–I temperature. For both 0.1 wt % and 0.3 wt % Au NP concentrations, the phase transition temperature is close to that of an undoped LC (for the LC cell and MC) and more noticeable changes are visible only for higher concentrations. For a PLCF with 1 wt % Au NP concentration, the N–I phase transition temperature decreased from 43.2 °C to 40.1 °C. A possible explanation is that with the general reduction of the N–I temperature, the higher NP concentrations start to create clusters and hence the nematic LC phase is disrupted. In [34], numerical calculations confirmed the possibility of lowering the N–I phase transition temperature for a host material with metallic NPs.

**Table 1:** Comparison of phase transition temperatures for three setup structures (liquid crystal (LC) cell, microcapillary (MC), and photonic liquid crystal fiber (PLCF)) infiltrated with different concentrations of Au-doped liquid crystal. The lowest obtained transition temperatures for 6CHBT with 1.0 wt % Au concentration are in bold font.

Infusion material	*T*_N–I_ for LC cell [°C]	*T*_N–I_ for MC [°C]	*T*_N–I_ for PLCF [°C]

6CHBT	43.7	43.7	43.2
0.1 wt % Au + 6CHBT	43.5	43.5	41.6
0.3 wt % Au + 6CHBT	43.2	43.2	41.5
0.5 wt % Au + 6CHBT	42.4	42.9	41.3
1.0 wt % Au + 6CHBT	**41.8**	**42.0**	**40.1**

### Switching times under an external electric field

In this part we investigated the influence of gold NPs on switching time under the action of an external electric field. Preliminary experiments on this subject were performed for a LC cell with a Ti-doped 5CB [18] and the obtained results showed that the switching times (10–20 ms) are almost independent of the presence of NPs. The fact that in both cases the switching times are comparable may be related to some of the parameters of the 5CB nematic LC (such as chemical structure, composition or stability). For this reason in further experiments we replaced the 5CB LC with the 6CHBT LC and we focused on the PLCF rather than the LC cells.

**Figure 12:**
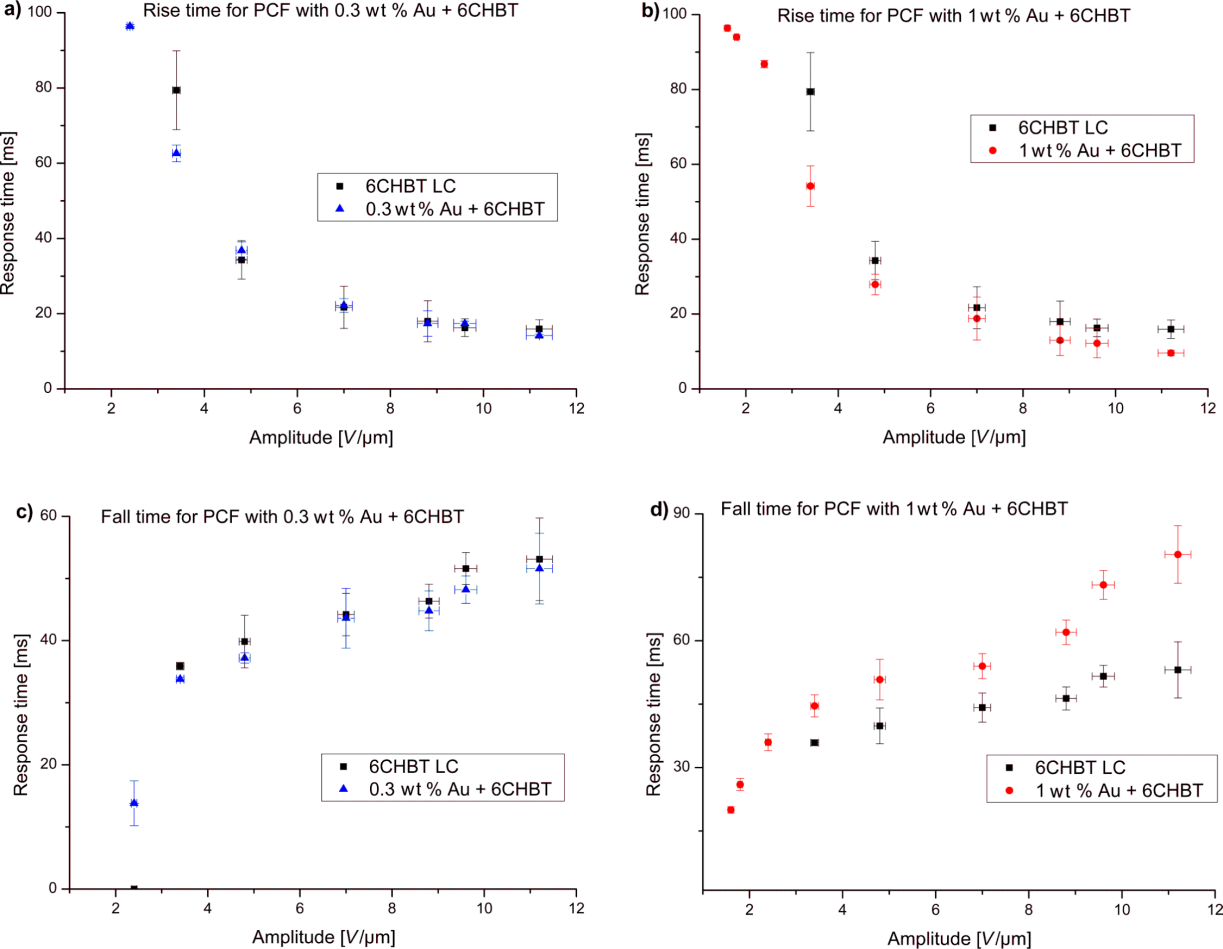
Rise time (a,b) and fall time (c,d) for 0.3 wt % Au and 1 wt % Au-doped photonic liquid crystal fibers compared to undoped LC 6CHBT.

**Figure 13:**
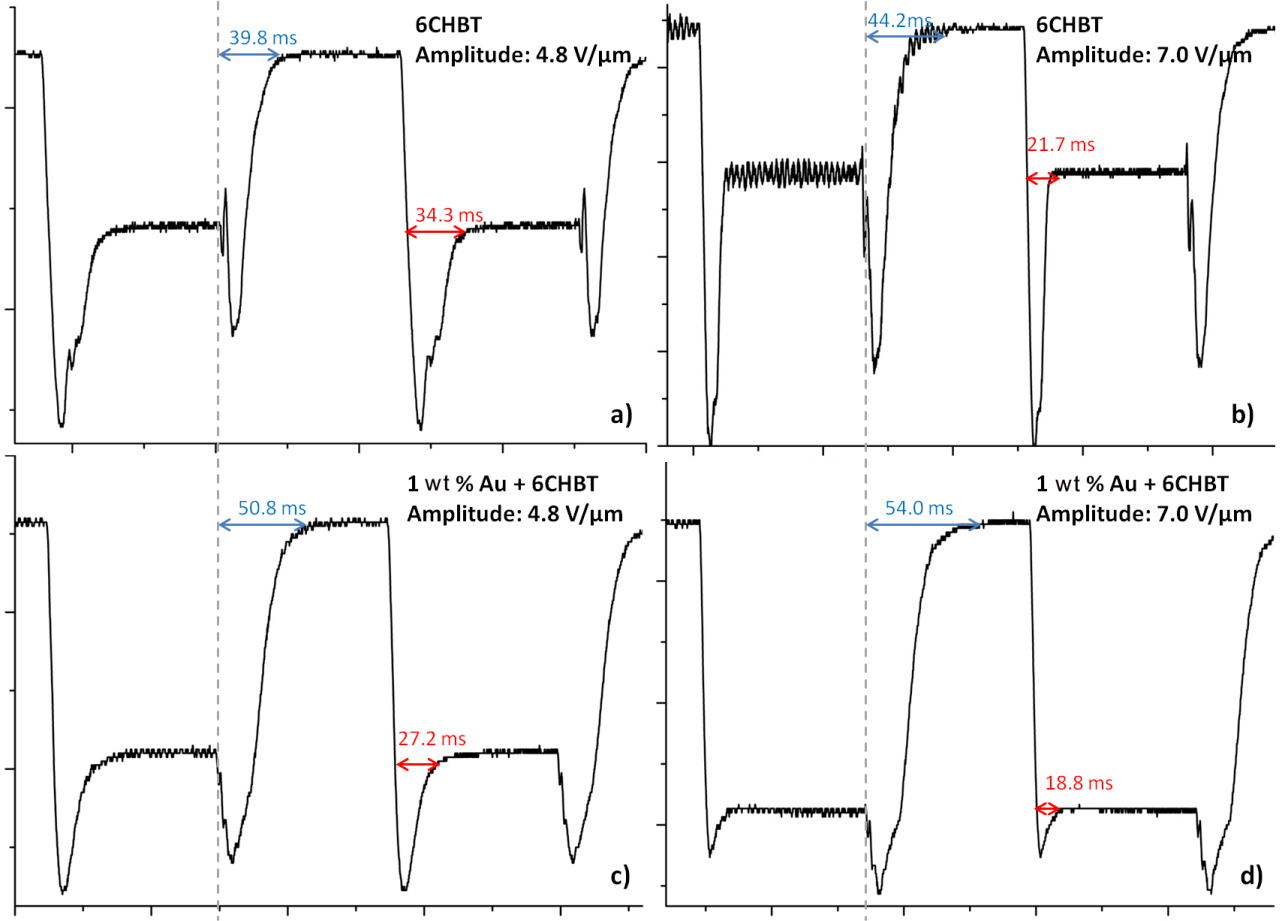
Selected oscillograms for undoped (a,b) and 1 wt % Au-doped (c,d) photonic liquid crystal fibers for two amplitudes: 4.8 V/µm and 7.0 V/µm. The red color was used to mark the rise time and the blue color for the fall time.

The obtained response time for samples with different concentrations are compared in Figure 12 and Figure 13; selected response times are also presented in Table 2. For all concentrations the experiment was repeated several times and results present average values of obtained switching times. For lower values of an electric field there is no or a very small change in switching time (lower than 10%). Moreover, with higher amplitude of the applied electric field the difference between undoped and doped LCs becomes significant. This difference is especially visible for concentrations of 0.5 wt % and 1 wt %. However, for fall times, the situation is reversed – low concentrations have similar switching times to an undoped LC, but for higher NP concentrations, LC molecules need more time to return to their original position. For an electric field intensity of about 8.0 V/µm and higher, the relaxation time could exceed even the times for an undoped LC. This effect could be connected with the electric constant of metallic dopants. High concentrations of Au NPs give good response times (25–30% shorter than for the undoped LC), but the relaxation times reduce this effect with an increase of electric field intensity. This could not be attributed to ionization of the LC (due to the use of an AC field) since Au NPs are considered as ion-capturing nanomaterials (along with carbon nanotubes, isolating and ferroelectric NPs [35]). In addition, the effect alone would be negligible due to the size of the Au NP dopants (about 6 nm) as well as their distribution in microcapillaries of the PCF. However, the reported noticeable ionization was for NPs of size larger than 50 nm, where there could be an increased charge density at the surface of the NPs, affecting the behavior of LC molecules and thus more time would be required for molecules to return to their original state. A similar response to the external electric field was observed in Au-doped nematic E7 LC in a LC cell [36], where additionally Hsu et al. described the reduction of both nematic–isotropic phase transition temperature and threshold voltage. The latter effect also confirms our observations. As can be seen in Table 2, where the presence of metallic NPs reduced the threshold voltage needed to reorient the LC molecules. For undoped PLCFs, the molecules start switching above 2.4 V/µm and for Au-doped LCs, the threshold lowers to 1.8 V/µm or even 1.6 V/µm (for 1 wt % Au concentration). It is worth emphasizing that for lower electric fields there is a sufficient reduction in the rise time and simultaneously the lowest increase in fall time. We would like to emphasize that in the Au-doped PLCF, the threshold voltages are significantly lower than in undoped PLCFs, which is crucial for this type of photonic device.

**Table 2:** Selected values of rise and fall times for undoped and Au-doped photonic liquid crystal fibers. The biggest differences between switching times for undoped and Au-doped photonic liquid crystal fibers are highlighted in bold font.

	Rise time [ms]					Fall time [ms]				
Amplitude [V/µm]	6CHBT	0.1 wt % Au	0.3 wt % Au	0.5 wt % Au	1.0 wt % Au	6CHBT	0.1 wt % Au	0.3 wt % Au	0.5 wt % Au	1.0 wt % Au

1.6	–	–	–	–	96.4	–	–	–	–	20.4
1.8	–	–	–	95.6	94.0	–	–	–	12.0	26.0
2.4	–	95.4	96.4	95.4	86.8	–	10.4	13.8	16.2	36.0
3.4	79.4	66.6	62.6	62.0	54.2	35.9	30.2	33.8	34.6	44.6
4.8	34.3	34.0	36.8	32.1	27.9	39.8	37.8	37.2	43.4	50.8
8.8	18.0	18.8	17.4	16.8	13.0	46.3	40.0	44.8	49.0	62.0
11.2	**15.9**	16.0	**14.2**	**12.2**	**9.6**	**53.1**	49.7	51.6	**56.0**	**80.4**

The influence of a gold NP on the electro-optical properties of a host material has been known for some time, but it is still uncertain what the real origin of the effect is. We attribute this behavior to one of two explanations presented in literature. In some of the papers [26,37], this is due to the intrinsic electrical properties of NPs, which after coupling with an LC director, pass their strong reaction to external stimulation onto the host material. On the other hand, in [38], the reaction of an LC–NP composite to the electric field is related to the order parameter. The reduction of the order parameter can be confirmed by two effects observed during our experiments. The first one is the lowering of the threshold voltage presented in Table 2 and the second is the reduction of the nematic–isotropic phase transition temperature visible in Figures 7–11. Both of these effects indicate that the reduction of the elastic constant occurs as well as the reduction of the order parameter (as the elastic constant is proportional to the square of the order parameter). This is also responsible for the improvement of the response time of the measured PLCF sample. On the other hand, the increase of the fall time with a higher concentration of NPs can be related to hindering of the NP LC reorientation. The presence of a high concentration of NPs could locally disturb the orientation of the LC molecules, resulting in a longer time needed for molecules to return to their original position.

## Conclusion

In conclusion, there is a noticeable difference between the response to external stimulation for undoped and NP-doped LCs. At room temperature we have observed different PBGs in the spectrum, depending on which material was used to infiltrate the PLCF. For higher dopant values, there is a visible shift towards longer wavelengths in the light spectrum. After heating the samples, a reduction of the N–I phase transition temperature for all three setup structures was observed. Finally, the experiments conducted in the presence of an external electric field confirmed that gold NPs lower the switching (rise) time as well as the threshold voltage in PLCFs. We assume that these parameters have been improved due to the fact that the NPs diameter (6 nm) is comparable to the 6CHBT molecules (about 2.6 nm). Summarizing, the results are very promising and suggest further improvement of the response times for NP-doped PLCFs. The obtained results can be used to enhance the electro-optical properties of the PLCF-based devices where the response time is a crucial issue.
